# ASCO 2020: highlights in breast cancer

**DOI:** 10.1007/s12254-021-00674-9

**Published:** 2021-01-24

**Authors:** Rupert Bartsch

**Affiliations:** Department of Medicine 1, Division of Oncology, Medical University of Vienna, Waehringer Guertel 18–20, 1090 Vienna, Austria

**Keywords:** ASCO Annual Meeting 2020, Highlights, Pembrolizumab, Review, Tucatinib

## Abstract

The 2020 Annual Meeting of the American Society of Clinical Oncology (ASCO) was held in a virtual format due to the ongoing SARS-CoV‑2 pandemic. Despite these unique circumstances, results of several interesting studies in the field of breast cancer (BC) were reported. While overall survival data are still missing, KEYNOTE-355 suggests significant activity of pembrolizumab when added to first-line chemotherapy in metastatic triple-negative breast cancer. TBCRC 048 evaluated the role of olaparib in homologous recombination deficient tumours due to genomic alterations other than germline *BRCA1/2* mutations; clinically relevant activity was reported in patients with germline *PALB2* and somatic *BRCA1/2* mutations. In HER2-positive early stage disease, different strategies of chemotherapy de-escalation are under investigation, but the optimal approach is still not well defined. Updated results from the HER2CLIMB trial show that the third-generation HER2 tyrosine-kinase inhibitor tucatinib in combination with trastuzumab and capecitabine is the new standard-of-care for pretreated patients with HER2-positive metastatic BC with active brain metastases. Results from BYLieve supports the notion that the combination of endocrine therapy with the PIK3Ca inhibitor alpelisib is a reasonable treatment approach in hormone-receptor positive/HER2-negative BC after prior CDK4/6-inhibitor therapy. Finally, the ECOG-ACRIN 2108 trial failed to show a benefit for early surgery of the primary tumour in patients with metastatic BC.

## Introduction

Despite being unique due to its virtual format, results of several clinically important trials were presented at this year’s American Society of Clinical Oncology (ASCO) Annual Meeting in the field of breast cancer (BC). This article is intended as a short review of these data.

## KEYNOTE-355

Based upon results from the prospective randomized phase III trial IMpassion130, the combination of nab-paclitaxel and the PD-L1 (programmed death-ligand 1) inhibitor atezolizumab is regarded as the standard-of-care for the first-line treatment of PD-L1 positive metastatic triple-negative breast cancer (mTNBC) [1]. KEYNOTE-355 evaluated the PD‑1 inhibitor pembrolizumab in combination with chemotherapy (paclitaxel, nab-paclitaxel or carboplatin/gemcitabine) in the same setting [2]; in total 847 patients ware accrued to this randomized placebo-controlled phase III trial. Co-primary endpoints were progression-free survival (PFS) and overall survival (OS) in the PD-L1 positive population (defined as combined positive score [CPS] ≥ 10 and ≥ 1) and the intent-to-treat (ITT) population. In contrast to IMpassion130, patients with a disease-free interval since the end of adjuvant chemotherapy of 6–12 months could enrol.

In the CPS ≥ 10 cohort (38.9% and 36.7% of patients in the pembrolizumab and placebo group, respectively), a significant prolongation of PFS from 5.6 to 7.6 months was observed (HR 0.74, 95% CI 0.61–0.90, *p* = 0.0014); in the CPS ≥ 1 and the ITT population, no significant improvement was reported. The PFS benefit was observed across all subgroups but appeared to be less pronounced in the group of patients with early relapse although confidence intervals overlap, and this apparent difference may have been caused by small patient numbers in this subgroup. Treatment-related adverse events (AEs) were comparable, with the expected exception of immune-related AEs.

These data support the role of immunotherapy as first-line treatment in combination with chemotherapy in PD-L1 positive mTNBC. OS data of KEYNOTE-355 have not yet been presented yet, and these must be awaited before approval of pembrolizumab can be expected. In the light of recent negative results obtained with the combination of paclitaxel and atezolizumab (IMpassion131) [3], the differential effects of pembrolizumab when added to paclitaxel or nab-paclitaxel needs to be analysed in more detail and these data are eagerly awaited.

## TBCRC 048

Targeting homologous recombination deficiency (HRD) is a promising treatment approach and the poly (ADP-ribose) polymerase (PAPR) inhibitors olaparib and talazoparib have been approved meanwhile for the treatment of metastatic breast cancer (mBC) patients harbouring *BRCA1/2* germ-line mutations. Other genomic alterations, however, may also result in homologous recombination deficiency (HRD) but little is known about the potential activity of PARPi in these tumours.

TBCRC 048 evaluated the activity of olaparib in mBC patients with germline mutations associated with HRD other than *BRCA* (cohort 1) and somatic mutations including somatic (s) *BRCA1/2* mutations (cohort 2) [4]. In total, 54 pretreated patients (75% ER-positive/HER2-negative; 19% TNBC) were accrued; the most common genomic alterations were mutations of *ATM, CHEK2, PALB2* and somatic mutations of *BRCA1/2*. In cohort 1, a response rate of 33% was reported; all responses were seen in patients with germline *PALB2* mutations and the response rate in this subgroup was 82%. In cohort 2, response rate was 31% with all confirmed responses observed in *sBRCA* mutant tumours; response rate in this subgroup was 50%.

While these data are clearly preliminary, results or TBCRC 048 suggest that PAPR-inhibitors may have a role beyond germline *BRCA* mutation in breast cancer.

## HER2-positive early breast cancer

In early stage HER2-positive breast cancer, the scientific focus is currently on de-escalation strategies. Neoadjuvant chemotherapy in combination with the HER2-directed antibodies trastuzumab and pertuzumab is regarded as the standard-of-care for stage II and III HER2-positive disease [5]. It is, however, unclear if all patients require full-course anthracycline- and taxane-containing chemotherapy.

The PHERGAIN trial investigated a response-adapted approach based upon metabolic response to antibody therapy [6]. In total, 356 patients were accrued and randomized to a standard arm consisting of six cycles of docetaxel, carboplatin and trastuzumab/pertuzumab (TCHP) or an experimental arm of TP without chemotherapy; at baseline and after two cycles, a PET-CT was conducted. In metabolic responders in the experimental arm (defined as a maximum SUV reduction of ≥ 40%), immunotherapy was continued for another six cycles followed by surgery. In non-responders, 6 cycles of TCHP were administered. Co-primary endpoints were pathologic complete response (pCR) rate in PET-responders and 3‑year invasive DFS (iDFS) in the experimental arm.

In the experimental arm, 79.6% of patients were regarded as PET-responders; in this group, a pCR rate of 37.9% was achieved (95% CI 31.6–44.5) rejecting the null hypothesis that pCR rate without chemotherapy was ≤ 20%. Of note, pCR rate were numerically higher in patients with HER2 3+ overexpressing tumours as opposed to 2+/ISH positive BC (40.8% vs. 25.6%) and among the highest chemotherapy-free pCR rates ever reported in HER2-positive BC. While this de-escalation approach is highly attractive due to the reduction of side effects, iDFS results must be awaited before translation into clinical practice can be considered.

The phase III TRAIN‑2 trial evaluated the activity of an anthracycline-free chemotherapy backbone; 438 patients were accrued and randomized to 9 cycles of paclitaxel, carboplatin, trastuzumab and pertuzumab or three cycles of FEC (5-FU, epirubicin, cyclophosphamide) + TP followed by six cycles of paclitaxel, carboplatin + TP. pCR rates have already been published and no differences between the two arms were observed (pCR rate 68% and 67%, respectively) [7]. At this year’s ASCO Annual Meeting, long-term outcome data at a median follow-up of 3 years were presented [8]. As expected, event-free survival (EFS) was significantly better in patients achieving pCR (94.1% vs. 85.1%; HR 0.42; 95% CI 0.23–0.78) while no differences were observed in terms of EFS and OS between the two treatment arms. Regarding toxicity, a higher rate of febrile neutropenia and LVEF decline was observed in the anthracycline-containing arm, while the rates of peripheral sensory neuropathy were comparable.

While these data support the notion that anthracyclines may not be a mandatory component of neoadjuvant chemotherapy in the presence of dual HER2 blockade in HER2-positive disease, it must be pointed out that TRAIN‑2 in fact escalated the number of chemotherapy cycles in both arms.

## HER2CLIMB

In HER2-positive metastatic breast cancer, treatment standards after progression on trastuzumab, pertuzumab and T‑DM1 are still ill defined. In HER2CLIMB, heavily pretreated patients were randomized to trastuzumab plus capecitabine with or without tucatinib; this third-generation HER2-directed tyrosine-kinase inhibitor (TKI) has profoundly higher activity against HER2 than EGFR, resulting in a reduced diarrhoea rate compared to older TKIs such as lapatinib or neratinib. Initial results of this prospective randomized placebo-controlled phase II trial have already been published [9]: In the tucatinib group, a clinically relevant and statistically significant prolongation of PFS (7.8 vs. 5.6 months; HR 0.54; 95% CI 0.42–0.71; *p* < 0.001) and OS (21.9 vs. 17.4 months; 95% CI 0.66; 95% CI 0.50–0.88; *p* = 0.005) was observed. Of note, 291 of 612 participants (47.6%) had brain metastases (BM) at baseline; of these, 174 patients (59.8%) had active BM (i.e. newly diagnosed or progressing after prior local therapy), making this the largest population of active BM patients in a randomized trial to date; data on the activity of tucatinib in this subgroup were eagerly awaited.

In the overall BM population, intracranial PFS was prolonged from 4.2 to 9.9 months (HR 0.32; 95% CI 0.22–0.48; *p* < 0.0001) and OS from 12.0 to 18.1 months (HR 0.58; 95% CI 0.40–0.85; *p* = 0.005). When only considering patients with active BM, outcomes were comparable (intracranial PFS 4.1 vs. 9.5 months; OS 11.6 vs. 20.7 months [10]). Therefore, results of HER2CLIMB define a potential novel systemic treatment standard for the subset of patients with active BM without indication for immediate local therapy.

## BYLieve

The combination of endocrine therapy with inhibitors of the cyclin-dependent kinases (CDKs) 4 and 6 is firmly established as the standard upfront treatment approach for metastatic luminal/HER2-negative breast cancer; the SOLAR-1 study established the activity of alpelisib, an alpha-specific inhibitor of phosphatidylinositol 3‑kinase (PI3K), when added to fulvestrant in pretreated patients with tumours harbouring *PIK3Ca* mutations [11]. Only a small minority of patients in SOLAR‑1, however, had received prior treatment with a CDK4/6 inhibitor.

The phase II BYLieve study evaluated the combination of alpelisib with endocrine therapy (either fulvestrant or letrozole) in patients with *PIK3Ca* mutant luminal/HER2-negative breast cancer who had progressed on prior systemic therapy [12]. At this year’s ASCO Annual Meeting, results from cohort A were presented; this cohort comprised 121 patients with centrally confirmed *PIK3Ca* mutant tumours who had progressed on or after prior treatment including a CDK4/6 inhibitor. At 6 months, 50.4% patients were alive and without disease progression (primary endpoint); median PFS was 7.2 months (95% CI 5.6–8.3) and 70.1% of patients had a decrease of measurable lesions. No new safety signals were observed, with diarrhoea and hyperglycaemia being among the most common AEs. The rate of rash were effectively reduced by the use of prophylactic antihistamines.

Overall, efficacy data of cohort A of BYLieve compare well with SOLAR‑1, where 44.4% of patients where free of progression at 6 months. This suggests that alpelisib is a meaningful treatment option also in patients progressing on prior CDK4/6.inhibitor therapy.

## ECOG-ACRIN 2108

Several earlier studies suggested that mBC patients may benefit from surgical removal of the primary cancer. Three randomized trials, among them Austrian Breast and Colorectal Cancer Study Group trial 28 [13], however, yielded conflicting results with a Turkish study suggesting a potential benefit of surgery [14].

In ECOG-ACRIN 2108, 256 patients with mBC without disease progression after 4–8 months of systemic therapy were randomized to continued systemic therapy with or without additional early local therapy (ELT) [15]. The majority of patients had luminal/HER2-negative breast cancer, 37.9% presented with bone-only disease and 53.8% had received upfront chemotherapy. In the overall study population, no difference in terms of OS was observed (HR 1.09; 95% CI 0.80–1.49); in the subset of patients with mTNBC, additional ELT seemed to have a detrimental effect (risk for death HR 3.5; 95% CI 1.16–10.57). Therefore, additional locoregional therapy may not be regarded as a standard component of mBC treatment.

### Take-home message

Addition of immunotherapy to first-line chemotherapy is active in PD-L1 positive mTNBC patients but OS data of KEYNOTE-355 are required to allow for a full appraisal of the role of pembrolizumab. For now, atezolizumab plus nab-paclitaxel remains the standard-of-care in this setting. In early stage HER2-positive breast cancer, trials exploring different de-escalation strategies were presented; the PHERGAIN study evaluating response-adapted treatment is promising, but data on long-term outcomes are still awaited. In HER2-positive metastatic disease, the combination of tucatinib, trastuzumab and capecitabine defines a novel treatment standard for pretreated patients with active brain metastases. Finally, BYLieve supports the notion that the PIK3Ca inhibitor alpelisib is a reasonable treatment option in patients with *PIK3Ca* mutant luminal breast cancer progressing on CDK4/6-inhibitor therapy.
